# Spectroscopic determination of leaf chlorophyll content and color for genetic selection on *Sassafras tzumu*

**DOI:** 10.1186/s13007-019-0458-0

**Published:** 2019-07-11

**Authors:** Yanjie Li, Yang Sun, Jingmin Jiang, Jun Liu

**Affiliations:** Research Institute of Subtropical Forestry, Chinese Academy of Forestry, No. 73, Daqiao Road, Fuyang, Hangzhou, 311400 Zhejiang Province China

**Keywords:** NIR spectroscopy, Prediction, *Sassafras tzumu*, Genetic selection

## Abstract

**Background:**

Reflectance spectroscopy, like IR, VIS–NIR, combined with chemometric, has been widely used in plant leaf chemical analysis. But less studies have been made on the application of NIR reflectance spectroscopy to plant leaf color and pigments analysis and the possibility of using it for genetic breeding selection. Here, we examine the ability of NIR reflectance spectroscopy to determine the plant leaf color and chlorophyll content in *Sassafras tzumu* leaves and use the prediction results for genetic selection. Fresh and living tree leaves were used for NIR spectra collection, leaf color parameters (a*, b* and L*) and chlorophyll content were measured with standard analytical methods, partial least squares regression (PLSR) were used for model construction, the coefficient of determination (R^2^) [cross-validation (${\text{R}}^{2}_{\text{CV}}$) and validation (${\text{R}}^{2}_{\text{V}}$)] and root mean square error (RMSE) [cross-validation (RMSE_CV_) and validation (RMSE_V_)] were used for model performance evaluation, significant Multivariate Correlation algorithm was applied for model improvement, to find out the most important region related to the leaf color parameters and chlorophyll model, which have been simulated 100 times for accuracy estimation.

**Results:**

Leaf color parameters (a*, b* and L*) and chlorophyll content were well predicted by NIR reflectance spectroscopy on fresh leaves in vivo. The mean ${\text{R}}^{2}_{\text{CV}}$ and RMSE_CV_ of a*, b*, L* and chlorophyll content were (0.82, 4.43), (0.63, 3.72), (0.61, 2.35) and (0.86, 0.13%) respectively. Three most important NIR regions, including 1087, 1215 and 2219 nm, which were highly related to a*, b*, L* and chlorophyll content were found. NIR reflectance spectra technology can be successfully used for genetic breeding program. High heritability of a*, b*, L* and chlorophyll content (*h*^2^ = 0.77, 0.89, 0.78, 0.81 respectively) were estimated. Several families with bright red color or bright yellow color were selected.

**Conclusions:**

NIR spectroscopy is promising for the rapid prediction of leaf color and chlorophyll content of living fresh leaves. It has the ability to simultaneously measure multiple plant leaf traits, potentially allowing for quick and economic prediction in situ.

## Background

Reflectance spectroscopy combined with advanced chemometric modelling methods has been successfully used as a rapid and effective method to estimate the chemical and pigment components in leaves [1–3]. However, the application of field-based spectroscopy to assess the pigment of living leaves in situ has lagged.

Chlorophyll, carotenoid and anthocyanin are the three most important pigments in leaves [4]. Chlorophyll, commonly responsible for green color, is an essential pigment for the conversion of light into chemical energy [5]. Carotenoid is mainly related to the yellow color during the chlorophyll degradation, and the increased synthesis of anthocyanins is the main reason leading to red. The proportion of these pigments in leaf changes in autumn, as a result of different degradation degrees of chlorophyll and carotenoids and the synthesis of anthocyanin, contributing to a high ornamental value [6]. Chlorophyll mainly determines the photosynthetic rate and primary productivity in plant and is widely used as a response to the environment stress and nitrogen fertilizer application. Chlorophyll content will be changed with the change of external environment, which could further lead to the photosynthetic capacity change [3]. Therefore, chlorophyll content could be used as an important diagnostic indicator for plant growth study [7].

Leaf color and chlorophyll content play a critical role in plant growth and contribute greatly to the appearance of plants [8]. However, less work has been done in improving leaf color properties. The variation of color and chlorophyll is partly controlled by genes [9–11]. To ensure a quality and stable leaf color, it is necessary to reduce this variation, which can be achieved with genetic breeding selection program. However, genetic selection usually relies on a great sample size and a large scale of experiment [12, 13]. The assessments of chlorophyll contents are based on the extraction of chlorophyll with solvents from the destructive leaf followed by spectrophotometric determination [14]. The conventional method to obtain leaf color is by determining the value of three variables: L* (Lightness), a* (redness) and b* (yellowness) from laboratory CIELAB color system [15]. These methods are time and cost consuming and require labor in laboratory, not suitable for genetic selection. In contrast, field-based spectroscopy, offering rapid and non-destructive determination of these compounds in living leaves in situ, could be an effective way to reduce the need of a large number of sample collection in field, save the time and cost spent in analysis and allow for the assessment of a large number of individuals in a timely manner [16–18].

Near infrared (NIR) spectroscopy is a common reflectance spectroscopy frequently used in plant chemical estimation. It mainly relies on the vibrational excitation of three primarily molecular bonds from biochemical components, including C–H, N–H and O–H bonds, which results in variable absorption in NIR wavelength regions (700–2500 nm) [19]. To establish a reliable NIR prediction model, the individual chemical component which is measured by wet chemistry needs to be combined with reflectance spectra for model calibration using chemometric methods such as partial least squares regression (PLSR). Independent samples will be used for model validation and then the model could be used to predict the unknown samples by their reflectance spectra. NIR, with robust calibration and ability to screen large samples, has shown a reliable and promising ability in breeding selection programs [20].

*Sassafras tzumu*, wildly planted in the south of China, is a deciduous tree species with various and variable bright red or yellow leaf color changed in autumn. It is one of the most important colourful plants that could benefit the development of urban landscape [21]. However, the leaf color is unpredictable and has a large variation between red and yellow [22]. Little is known about the genetic variation of leaf color and chlorophyll contents in this species.

To uncover the crucial role that genetic variation plays in leaf color and chlorophyll content, it is important to develop a simple, nondestructive, real-time and intuitive approach for the measurement of leaf color and chlorophyll content. Here, we use field-based NIR spectroscopy to calibrate the leaf color and chlorophyll content prediction models in fresh leaves, which could provide a real-time and non-destructive estimation of the chemical components and allow for quick analysis of larger samples [13]. Specifically, we use NIR (1) to examine the quality of leaf color and chlorophyll content in fresh leaf, and (2) to estimate genetic parameters and correlations of leaf color and chlorophyll content.

## Results

### Color and chlorophyll content traits of *Sassafras tzumu* leaves

The a* and b* for the calibration data range from − 3.7 to 42.87 and 5.51 to 47.67 with CV of 0.55 and 0.39 respectively and 1 to 43.92 and 6.4 to 36.95 with CV of 0.52 and 0.41 for validation data respectively. L* has a small variation from 29.04 to 55.91 with an average of 35.82. Chlorophyll content has the highest coefficient of variation (0.56) compared to other traits, ranging from 0.16 to 3.87% with an average of 0.72% (Table 1).

**Table 1:** Summary statistics for the a*, b*, L* and chlorophyll content of *Sassafras tzumu* leaves in the calibration and validation data used for multivariate calibration of NIR spectra

	Calibration				Validation			
	Max	Mean	Min	CV	Max	Mean	Min	CV
a*	42.87	19.07	− 3.70	0.55	43.92	21.25	1.00	0.52
b*	47.67	15.95	5.51	0.39	36.95	17.17	6.40	0.41
L*	55.91	35.82	29.04	0.11	49.17	36.46	26.91	0.12
CC	3.87%	0.72%	0.16%	0.58	1.44%	0.60%	0.17%	0.56

*CV* coefficient of variation, *CC* chlorophyll content

### Model prediction

**Fig. 1:**
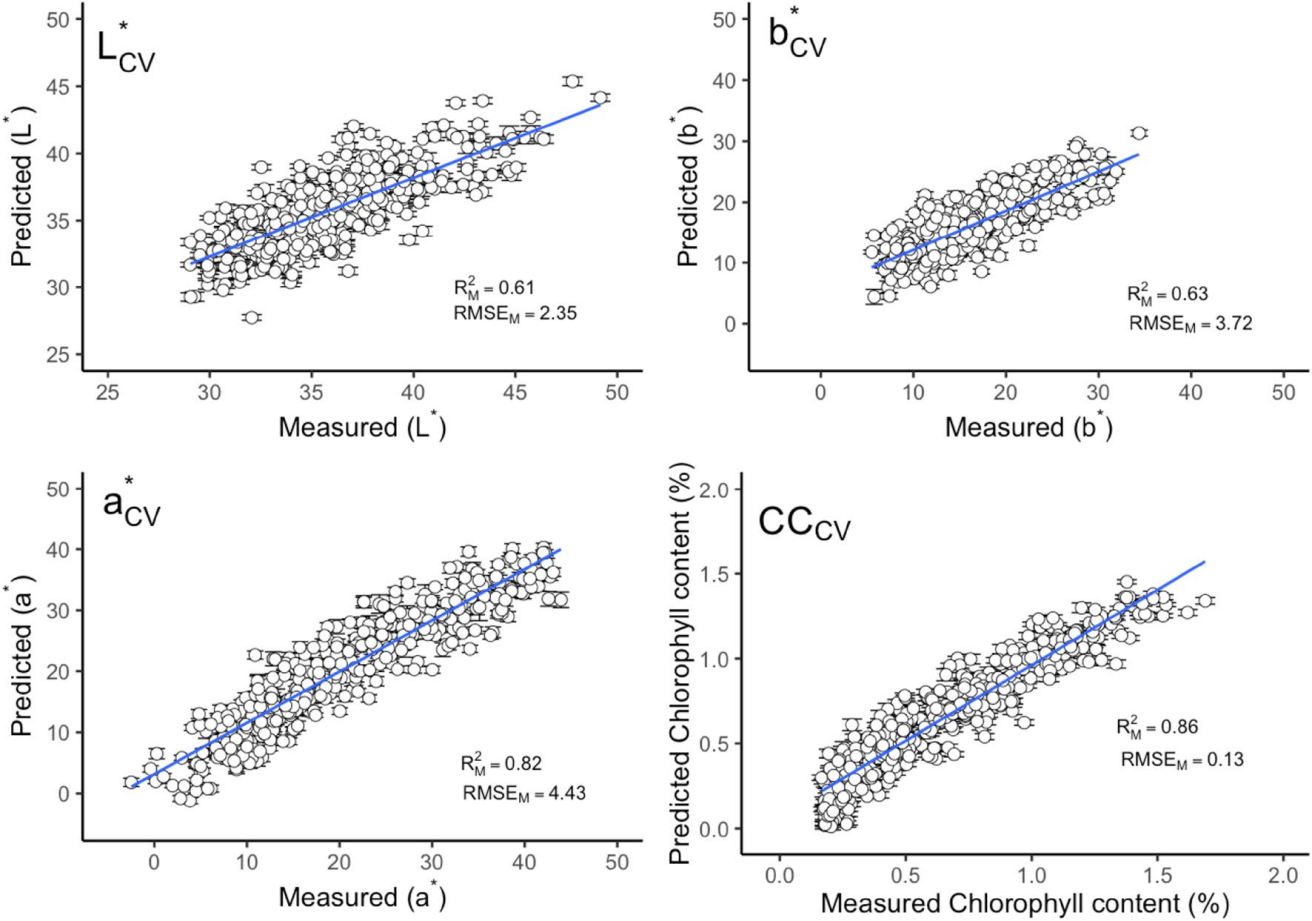
Measured versus predicted leaf color parameters and chlorophyll content in calibration data by full spectra model. Error bars for predicted values represent the standard deviations obtained from the 100 simulated models. ${\text{R}}^{2}_{\text{M}}$ and RMSE_M_: the mean value of coefficient of determination (R^2^) and root-mean-square error (RMSE) from 100 simulated models. *CC* chlorophyll content

**Fig. 2:**
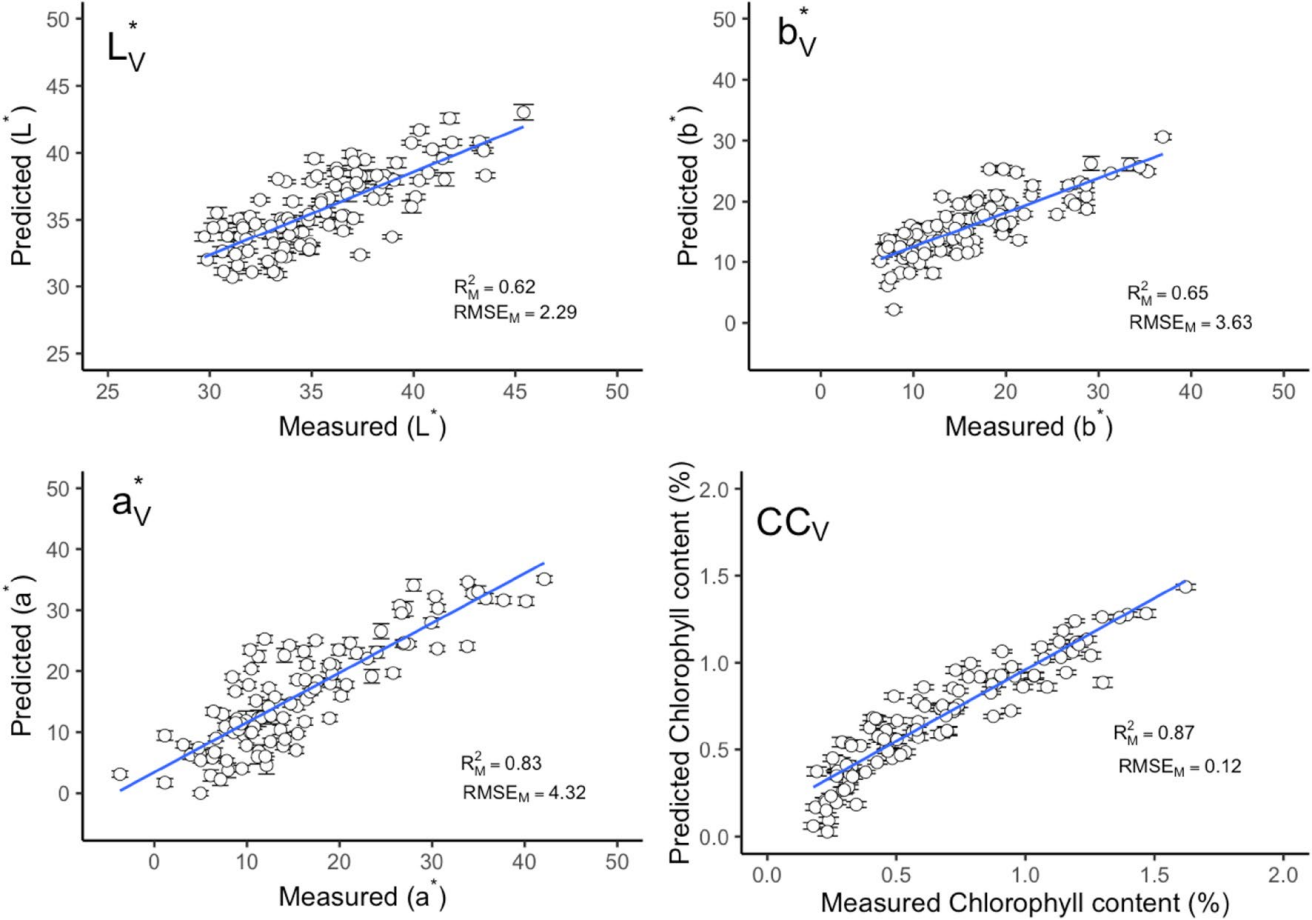
Measured versus predicted leaf color parameters and chlorophyll content in validation data by full spectra model. Error bars for predicted values represent the standard deviations obtained from the 100 simulated models. ${\text{R}}^{2}_{\text{M}}$ and RMSE_M_: the mean value of coefficient of determination (R^2^) and root-mean-square error (RMSE) from 100 simulated models. *CC* chlorophyll content

The leaf chlorophyll content and three different parameters of leaf color, i.e. a*, b* and L*, were considered as single prediction model separately. The best prediction model was found in chlorophyll content prediction with a mean ${\text{R}}^{2}_{\text{CV}}$ of 0.86 and RMSE_CV_ of 0.13%, followed by the a* prediction model (${\text{R}}^{2}_{\text{CV}}$ of 0.82, RMSE_CV_ of 4.43). The ${\text{R}}^{2}_{\text{V}}$ and RMSE_V_ of these two models have also shown reliable performance. The performance of b* and L* prediction models have shown less accuracy than chlorophyll content and a* model with a large variation of ${\text{R}}^{2}_{\text{V}}$ and RMSE_V_. The mean ${\text{R}}^{2}_{\text{CV}}$ and RMSE_CV_ of these two models are 0.63, 0.61, 3.72 and 2.35 respectively (Figs. 1 and 2).

### Variable selection and model optimization

**Fig. 3:**
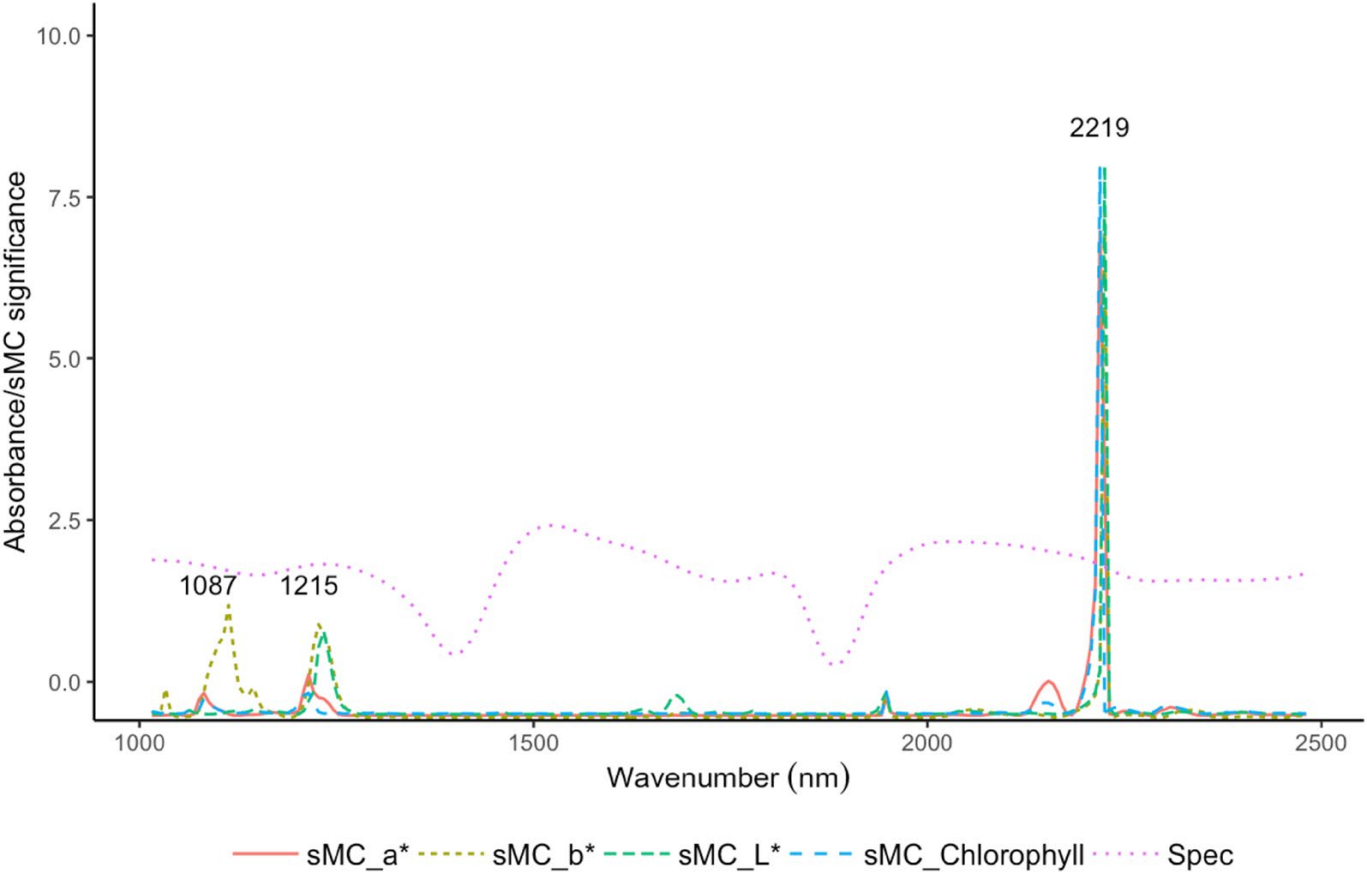
Influence of a*, b*, L* and chlorophyll content on NIR spectra in leaf of *Sassafras tzumu* model and the variable selected by the sMC algorithm

**Fig. 4:**
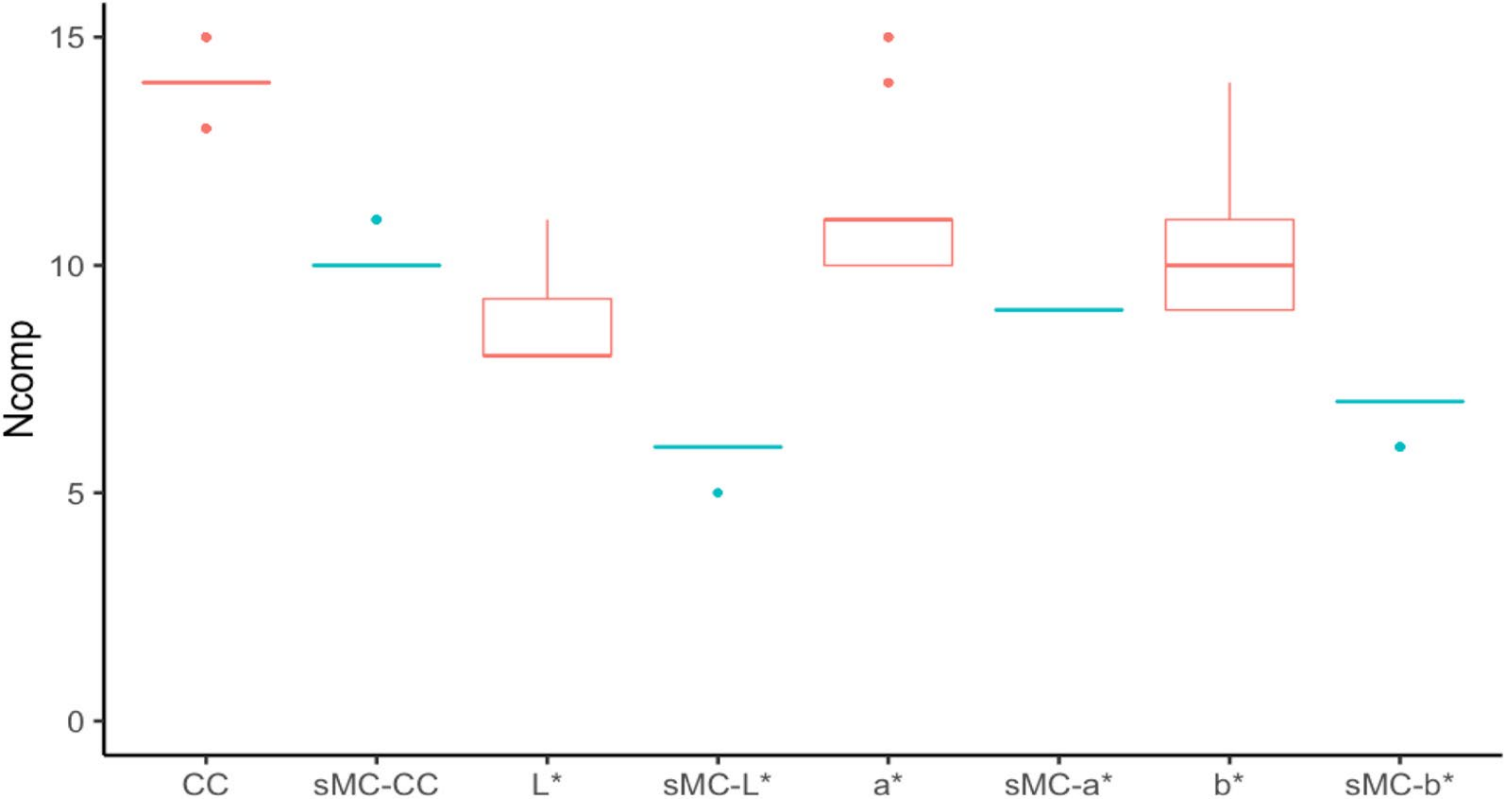
Optimal components range from the 100 simulated models for a*, b*, L* and chlorophyll content prediction in leaf of *Sassafras tzumu* with and without sMC variable selection. *CC* Chlorophyll content. Red color: without sMC variable selection, blue color: with sMC variable selection, solid line: mean optimal components, dot: outlier from the mean

**Fig. 5:**
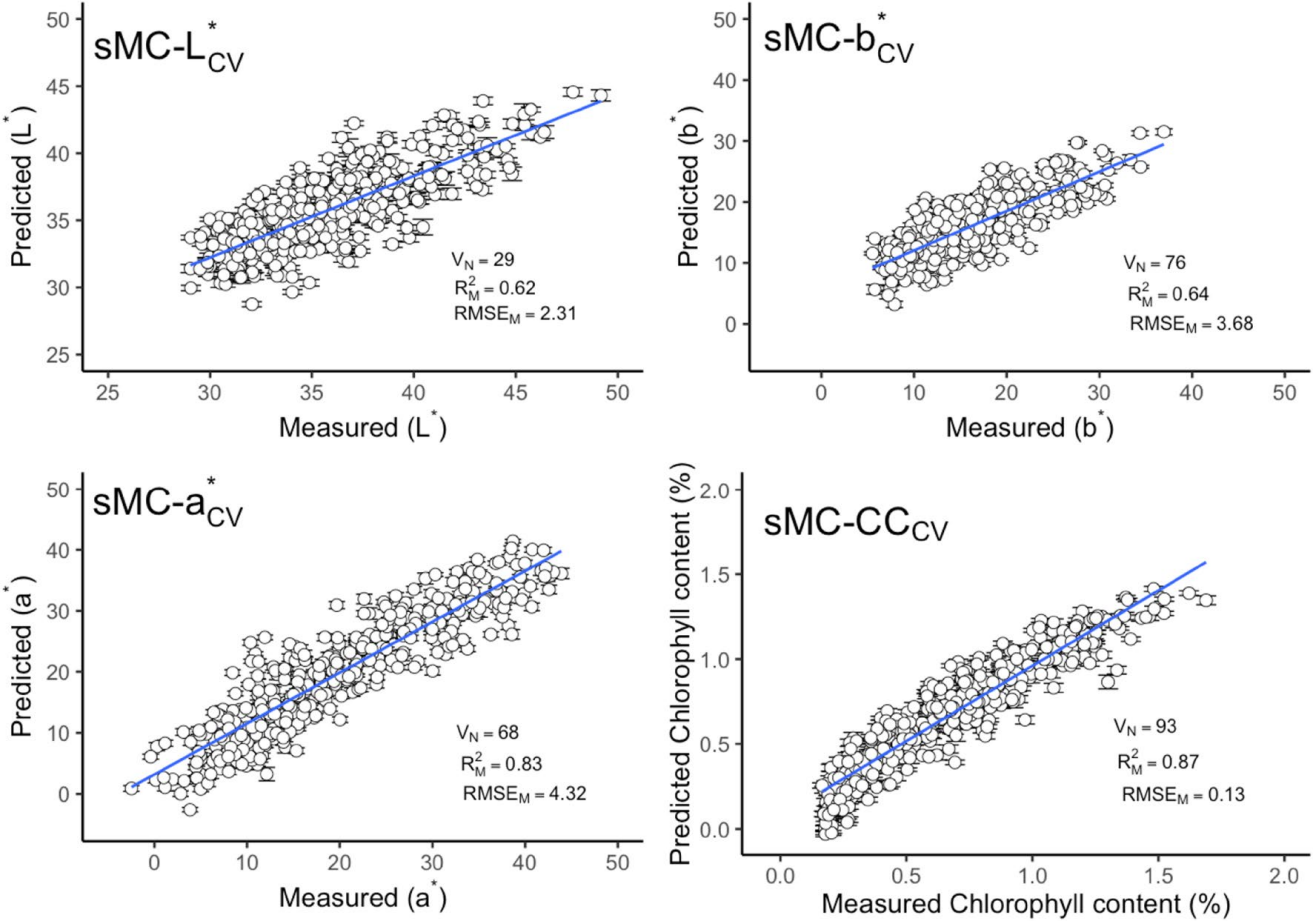
Measured versus predicted leaf color parameters and chlorophyll content in leaf of *Sassafras tzumu* from sMC calibration model. Error bars for predicted values represent the standard deviations obtained from the 100 simulated models. V_N_: Selected variable numbers. ${\text{R}}^{2}_{\text{M}}$ and RMSE_M_: the mean value of coefficient of determination (R^2^) and root-mean-square error (RMSE) from 100 simulated models. *CC* chlorophyll content

**Fig. 6:**
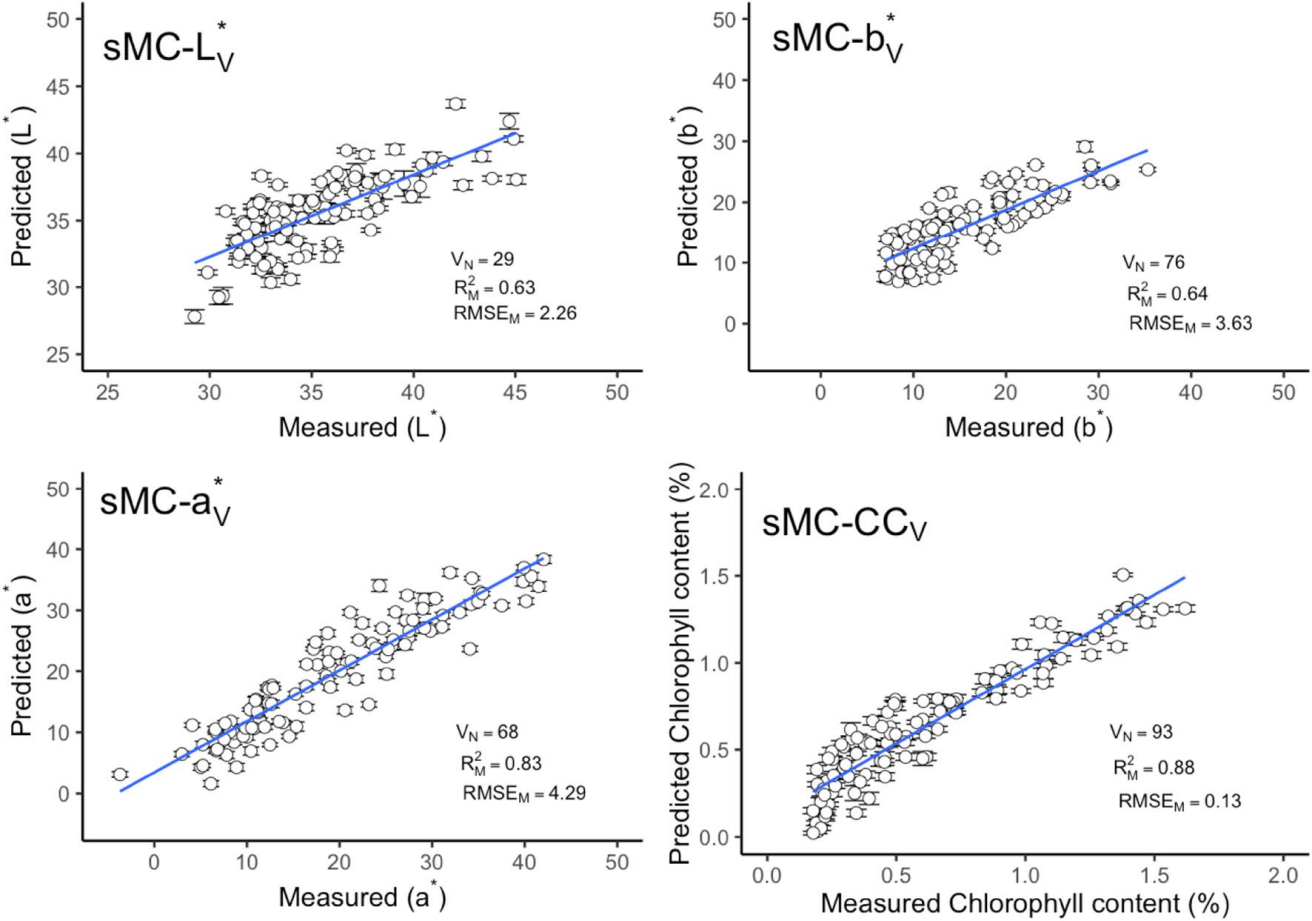
Measured versus predicted leaf color parameters and chlorophyll content in leaf of *Sassafras tzumu* validation data prediction by sMC model. Error bars for predicted values represent the standard deviations obtained from the 100 simulated models. V_N_: Selected variable numbers. ${\text{R}}^{2}_{\text{M}}$ and RMSE_M_: the mean value of coefficient of determination (R^2^) and root-mean-square error (RMSE) from 100 simulated models. *CC* chlorophyll content

The important variation that is highly linked to the observed trait could significantly influence the model prediction accuracy. In our study, we used sMC algorithm to find out the most useful information in the NIR spectra that highly contributed to the model prediction. Three most important regions in the NIR spectra, i.e. 1087, 1215 and 2219 nm, were similarly found in all four models. a*, b* and chlorophyll content shared the similar important regions in 1087 and 1215 nm while a*, b*, L* and chlorophyll content had the same selected region in 2219 nm (Fig. 3). The mean number of PLSR component for a*, b*, L* and chlorophyll content model were reduced from 11, 10, 8, 14 to 9, 7, 6 and 10 respectively (Fig. 4). Models with the application of sMC algorithm selection did not provide significant improvement on the model prediction accuracy, only slightly better than that of the full spectra models (Figs. 5, 6). However, compared to the full spectra models, sMC models use lesser components (Fig. 4) and highly reduce the number of spectra variables (reduce from full spectra numbers (242) to 68, 76, 29, 93 for a*, b*, L* and chlorophyll content model respectively) for model calibration (Figs. 5, 6).

### Heritability and phenotypic and genetic correlations among traits

High individual heritability was found in leaf color traits and leaf chlorophyll content (Table 1). The highest heritability was found in leaf b* value (*h*^2^ = 0.89), followed by chlorophyll content, L*, a* with *h*^2^ of 0.81, 0.78 and 0.77 respectively. Highest positive genetic and phenotypic correlations were found between L* and b* value (r_g_ = 0.93 and r_p_ = 0.90). The genetic and phenotypic correlations among b*, L*, a* were moderated ranging from 0.58 to 0.93. Chlorophyll content had a strong negative genetic and phenotypic correlation with a* (r_g_ = − 0.90, r_p_ = − 0.77), b* (r_g_ = − 0.75, r_p_ = − 0.57) and L*(r_g_ = − 0.72, r_p_ = − 0.52) (Table 2).

**Table 2:** The heritability, genetic (above diagonal) and phenotypic correlations (below diagonal) among traits with standard error between parentheses

Traits	a*	b*	L*	CC	*h* ^2^
a*		0.63 (0.11)**	0.58 (0.12)**	− 0.90 (0.03)***	0.77
b*	0.63 (0.04)**		0.93 (0.01)***	− 0.75 (0.08)***	0.89
L*	0.60 (0.04)*	0. 90 (0.01)***		− 0.72 (0.09)***	0.78
CC	− 0.77 (0.02)**	− 0.57 (0.04)*	− 0.52 (0.04)*		0.81

*CC* chlorophyll content, *h*^*2*^ heritability

*P ≤ 0.05, **P ≤ 0.01, *P* ≤ 0.001

### Family selection

**Fig. 7:**
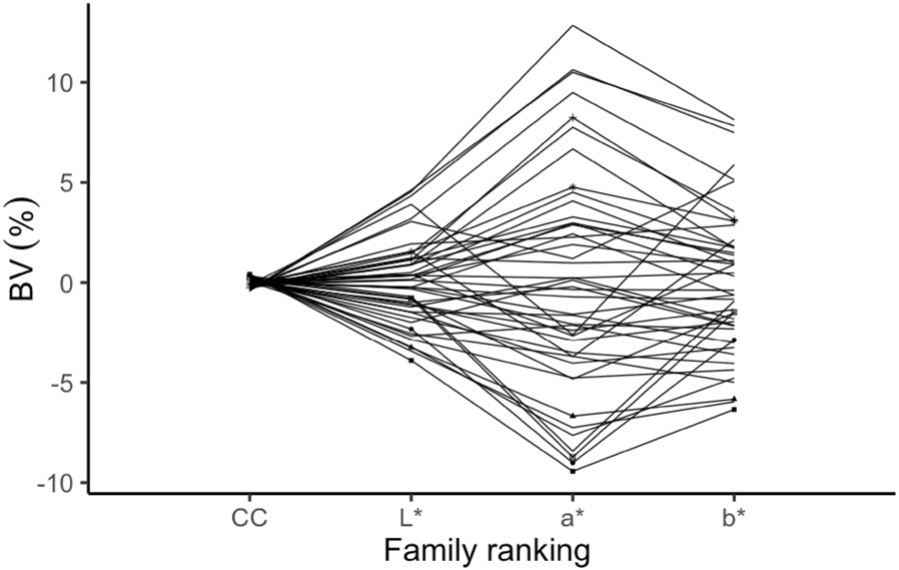
Family rankings for a*, b*, L*, chlorophyll content in *Sassafras tzumu* at age 2. Family values are expressed as deviation from each trait mean. *BV* breeding values, *CC* chlorophyll content

**Fig. 8:**
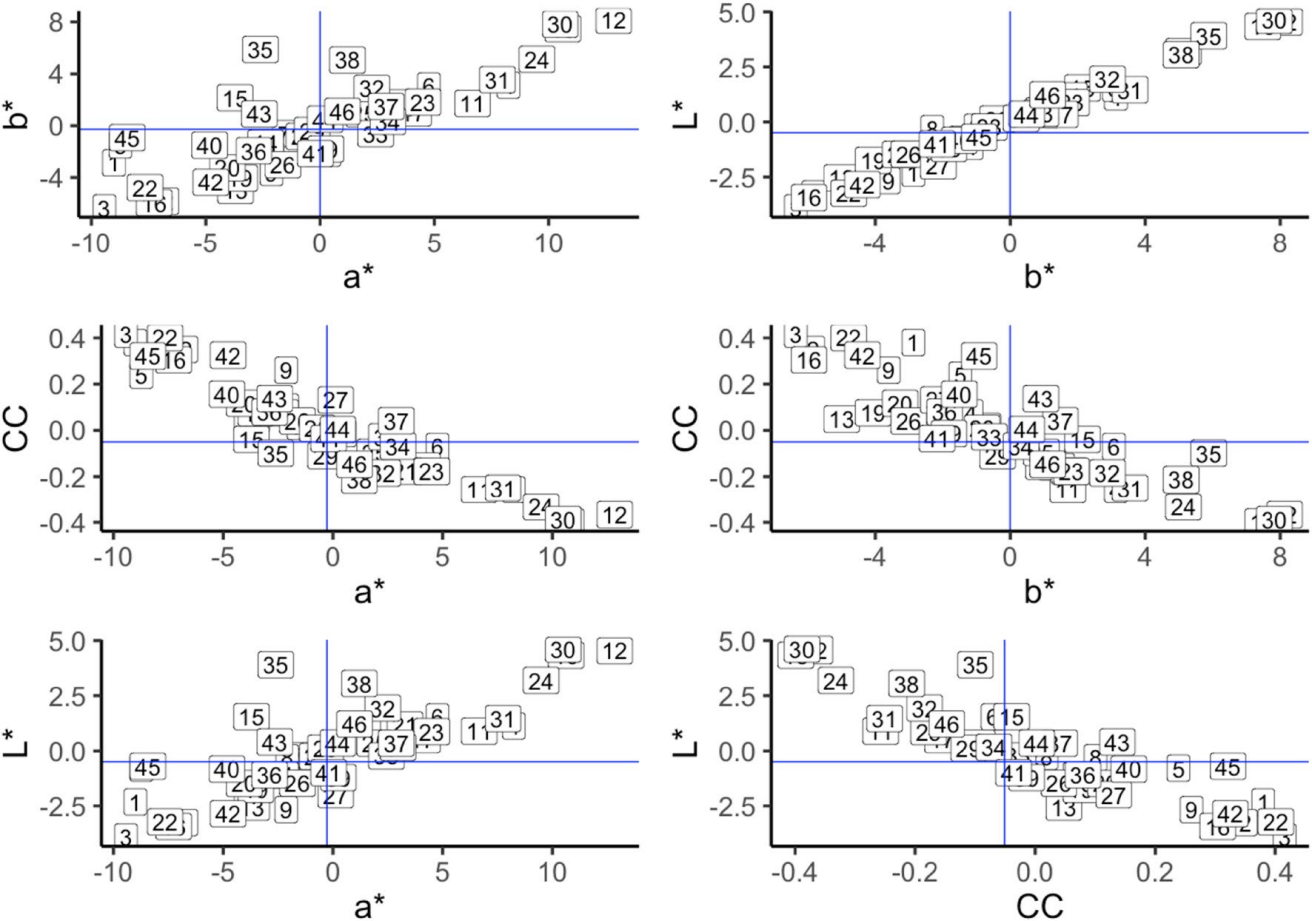
Relationship between a*, b*, L*, chlorophyll content breeding values of *Sassafras tzumu* families at age 2. *CC* chlorophyll content

Figure 7 displays the family ranking of breeding values for a*, b*, L* and chlorophyll content traits. The rankings between families were consistent across these four-leaf traits. It is possible to select traits according to certain purpose by families. The mean of a*, b*, L* and chlorophyll content relationship were plotted in Fig. 8. It is clear that some families could be used for various color selection. To lessen the green color influenced by chlorophyll content, less chlorophyll content and high color trait (a*, b*, L*) should be selected. Family 12, 23, 24, 30, 31, 32, 35, 38, 42, 46 and other 7 more could be selected for bright (high L*) red (high a* value) color breeding, while family 30, 31, 32, 35, 38, 44, 46 and other 10 more could be used for bright (high L*) yellow (high b* value) color selection.

## Discussion

The leaf chlorophyll content and color related traits in fresh leaves can be accurately predicted using field-base reflectance spectroscopy. The study, supported by Steidle Neto et al. [23, 24] and Xie et al. [24], presents a reliable and robust methodology on NIR reflectance spectra for all leaf traits to estimate the prediction model accuracy. This methodology was firstly reported by Couture et al. [13]. Compared with other sample selection methods, for instance, random selection [25] or Kennard-stone sample strategy [26], it could estimate the model uncertainty by providing the prediction error for each sample. The error bar could show the performance of model calibration and prediction (e.g. error bars in Figs. 1, 2, 5, 6). Compared with the standard color and chlorophyll content analyses, NIR reflectance spectroscopy is found to be a promising method for the leaf color and other pigments prediction.

It was reported that a small number of PLSR components could limit the outlier range and make the outside of calibration range still predictable without being classified as outliers [27]. Therefore, models with small number of components may yield better prediction results. The number of the PLSR components were significantly reduced after the sMC variable selection applied on the PLSR model. The sMC-PLSR models with a smaller number of components have shown a slightly higher prediction accuracy than that of the full spectra PLSR model.

Variation selection applied on the NIR spectra data could efficiently find the most important variables that highly related to the observation values. In our study, the important spectral features, including 1087, 1215 and 2219 nm, for the prediction of leaf a*, b*, L* and chlorophyll content, were found by the sMC selection algorithm. Supported by Datt [28] who found that the correlation between chlorophyll a content and NIR reflectance spectra in *Eucalyptus* leaf is higher in the range of 700–1300, 1500–1800 and 2100–2300 nm. Less studies are related to applying NIR on leaf color prediction, especially by using the wavelengths ranging from 1100 to 2500 nm. However, NIR has shown a promising performance on the prediction of wood color. The bands near 1112, 1784 nm are reported to be highly related to the color parameters (a*, and b*, L*) [29]. The band around 1087 nm and 1215 is mainly assigned to the second overtone of CH stretching vibration, while the band around 2219 nm is assigned to the CH stretching vibration [30].

The reflectance NIR spectroscopy in our study was applied on the living fresh leaves. One of the most disadvantageous aspects for spectra analysis on fresh leaf is water absorbance. Water with O–H bond has two significant absorbance regions (1414 and 1916–1980 nm) in the NIR spectra which may overlap other chemical information in the NIR spectra and lead to prediction model inaccuracy [30]. However, the three most important regions in NIR spectra that highly related to the leaf color parameter (a*, b*, and L*) and chlorophyll content are not overlapped with the water regions. The estimation of leaf color and chlorophyll content could promisingly be used on fresh leaves.

This field-based NIR reflectance technology is capable of fast and repeated leaf color estimation and leaf chlorophyll content analysis in vivo which is an important advancement to understand and develop knowledge of leaf ecology. Supported studies were found in fresh Ginkgo biloba leaves [31] and four common green-leafy species [32], which have shown that NIR reflectance technology could also be a reliable method to predict parameters and chlorophyll content for other species. Furthermore, leaf color and leaf pigment will respond to the stress and environment change, leaf color consistence and genetic variation. This repeatable and real-time spectral measurement could be used to track leaf color changes during the ecological variation.

For the half-sibling families selection, the 1/4 coefficient was usually used [33]. However, the coefficient of relationship in our study was used as 1/2.5, which is similar to the study reported by Li et al. [20], due to the unknown genetic structure of *S. tzumu*, the reproductive biology and population structure. Apiolaza [34] have used the coefficient between 1/3 and 1/2.5 to calculate deviations in the species which the family-level selling and spatially structured populations are not guaranteeing the deviations from 1/4. In addition, using different coefficients of relationship among siblings will not influence the genetic correlations [35].

It was reported that the use of low relatedness coefficients will result in the high individual heritability, which may be due to the assembling of half-siblings, inbreeding effects presented, and the same environment influence [20]. High individual heritability estimates were found for the leaf color parameters and chlorophyll content. The leaf color heritability in our study is higher than the results reported by Vogel et al. [36], who found a heritability of 0.59 for the leaf color of *Sorghastrum nutans* (L.). Nash. Townsend and McIntosh [37] also found that the parents have a significant influence on the leaf color of red maple (Acer rubrum L.). And relatively lower heritability were found for the *Oryza sativa* L (*h*^2^ ranges from 0.44 to 0.49) [38] and *Hevea brasiliensis* (*h*^2^= 0.22) [39] compared to the results in our study, suggesting the high potential for improving leaf color and chlorophyll content via selection for *S. tzumu*.

The leaf color parameters of a* and b* have a significant positive correlation with L*. L* value is the main determination of leaf color brightness. For various leaf color selection, it is vital to combine L* with the redness parameter a* or yellowness parameter b* for future selection. The family showed a consistent ranking for these four-leaf traits, suggesting that combining two more leaf traits for breeding selection are acceptable. Leaf color parameters have a significant negative correlation with leaf chlorophyll content. Chlorophyll mainly result in the leaf color being green, other pigments like carotenoid content mainly result in the leaf color being yellow and the anthocyanin content mainly result in the leaf color being red [40, 41]. Therefore, to select red or yellow leaf color, low chlorophyll content should be considered. Some families have been successfully selected for different selection targets, suggesting that various leaf color selection could be achieved by breeding selection program.

## Conclusion

Our results show that the field-based NIR reflectance spectroscopy can be a promising methodology for leaf color and chlorophyll content prediction and can be successfully used in genetic selection. It provides a promising and reliable capacity for other leaf pigments analysis in future. In addition, breeding selection methodology could be an efficient way to improve the leaf color quality.

## Methods and materials

### Leaf collections

A robust and accurate prediction model needs a large range of chemical variation. Therefore, we collected leaf samples from families with a wide range of color and chlorophyll content variation. 50 half-sib families, which were collected from 6 main different regions with high environmental variability in China, of 2 years old *S. tzumu* trees were selected in this study.

The trees were planted in 2016, Changle Forest Farm Nursery, Hangzhou, Zhejiang, China. Each family replicated 30 times. In October, when tree leaves changed color, 500 fresh leaves from 50 families were selected to calibrate NIR prediction model and other 1000 trees were used for genetic selection.

### NIR spectra collection

To reduce the color variation in tree level, for each tree, 5–6 leaves with similar color and on the same side were selected from the top to bottom and immediately collected the NIR reflectance spectra by using a wavelength field-based spectrometer (LF-2500, Spectral evolution, USA) with a handheld fibre optics contact probe. The probe was placed close to the leaf surface to avoid external light noise. Spectra was collected in a range of 1100 to 2500 nm with a 6 nm resolution and thirty-two scans were averaged for each leaf spectra. 500 tree leaves were immediately (within 1 day) collected to lab and placed in the refrigerator for chlorophyll content and color measurement.

### Leaf color measurement

Leaf color was measured using the CIELAB color system from a Minolta CM-3600A spectrophotometer (Konica, Japan). Each leaf was measured three times in three different surface positions and the average of the three variables L* (black to white (+)), a* (green to red (+)) and b* (blue to yellow (+)) were estimated.

### Leaf chlorophyll content measurement

A circular piece was cut from each leaf after NIR and color estimated for total chlorophyll content extraction, using a mortar to grand the leaf into powder and extracting with 100% acetone. The extracts were then centrifuged for 5 min in a glass tube and subsequently assayed by a UV–Visible spectrophotometer (UV-1280, Shimadzu, Japan). The equations and specific absorption in the wavelength reported by Wellburn [42] were used.

### Model calibration and validation

NIR spectra in our study were pre-processed by SNV + 2nd derivatives using Savitzky–Golay smoothing [43] with a window size of 17 data points. Partial least squares regression (PLSR) was used for model calibration using leave-one-out cross validation method. The coefficient of determination (R^2^) and root-mean-square error (RMSE) for both calibration and validation were used to track the model performance. Models were randomly performed 100 times using 80% of the data set for calibration and the remaining 20% for validation. The benefit of these randomized analyses was allowing for the assessment of the prediction model uncertainty and the overall model stability. R^2^ and RMSE were collected for each selection to assess the error of 100 calibration and validation model. The most important variables in the NIR spectra that highly explain the variation between variables and response chemical components were selected by using the filter method significant Multivariate Correlation (sMC) algorithm with a significance level of α = 0.05 [44]. This method is firstly estimating the variation of features from the PLSR model and then using these features to find out the significant feature for the PLSR model. The details of equation for sMC algorithm were described in other studies [44, 45].

### Statistical analysis

A multivariate restricted maximum likelihood (REML) linear mixed model was used for genetic parameter estimation. Single-trait observation y_i_ for a tree leaves was represented by the model:

1 $${\text{y}}_{\text{i}} = {\mathbf{x}}_{\text{i}} {\mathbf{m}} + {\text{f}}_{\text{i}} + {\text{e}}_{\text{i}}$$

**m**: fixed effect, **x**_i_: a vector linking the fixed effects **m** to the observation, *f*_i_, *e*_i_: the random family and residual effects.

Regarding the multivariate case, for each individual we have a vector of two observations **y**_i_ (phenotypes for trait 1, 2, 3…), and random vectors **f**_i_ and **e**_i_ for families and residuals. The model equation packed with those vectors for all tree leaves is as follow:

2 $${\mathbf{y}}_{ } = {\mathbf{Xm}} + {\mathbf{Z}}_{1} \varvec{f} + \varvec{e}$$

where **y**: a vector of phenotypic observations, **m**: the vector of fixed effects (overall mean), **f** and **e**: vectors of bivariate random effects for family and residual effects. **X** and **Z**_1_: incidence matrices linking observations to the appropriate effects.

The vector of expected values (E) and dispersion matrices (Var) were defined as: $E\left[ \varvec{y} \right] = {\mathbf{Xm}}$, $Var\left[ {\mathbf{f}} \right] = {\mathbf{Z}}_{1 \otimes } {\mathbf{F}}_{0}$, $Var\left[ {\mathbf{e}} \right] = {\mathbf{Z}}_{ \oplus } {\mathbf{R}}_{0}$, where $\otimes$ is the direct product operations and the $\oplus$ is the direct sum operations and

$${\mathbf{F}}_{0} = \left[ {\begin{array}{*{20}c} {\sigma_{f1}^{2} } & \cdots & {\sigma_{f1f4} } \\ \vdots & \ddots & \vdots \\ {\sigma_{f1f4} } & \cdots & {\sigma_{{f_{4} }}^{2} } \\ \end{array} } \right], {\mathbf{R}}_{0} = \left[ {\begin{array}{*{20}c} {\sigma_{e1}^{2} } & \cdots & {\sigma_{e1e4} } \\ \vdots & \ddots & \vdots \\ {\sigma_{e1e4} } & \cdots & {\sigma_{e4}^{2} } \\ \end{array} } \right],$$

where $\sigma_{{f_{i} }}^{2}$ and $\sigma_{{e_{i} }}^{2}$ represent the family and residual variances for trait $i$, and $\sigma_{fifj}$ and $\sigma_{eiej}$ are the family and residual covariances between traits $i$ and trait $j$. The narrow sense heritability $(h^{2}$) of trait $i$ and genetic correlations $\left( { r_{{g_{ij} }} } \right)$ and phenotypic correlation $( r_{{p_{ij} }} )$ between trait $i$ and trait $j$ were calculated as:

$$h_{i}^{2} = \frac{{2.5\sigma_{{f_{i} }}^{2} }}{{\sigma_{{f_{i} }}^{2} + \sigma_{{e_{i} }}^{2} }}$$

$$r_{{g_{ij} = }} \frac{{\sigma_{fifj} }}{{\sqrt {\sigma_{{f_{i} }}^{2} + \sigma_{{f_{j} }}^{2} } }}$$

$$r_{{p_{ij} = }} \frac{{\sigma_{fifj} + \sigma_{eiej} }}{{\sqrt {\left( {\sigma_{{f_{i} }}^{2} + \sigma_{{e_{i} }}^{2} } \right)\left( {\sigma_{{f_{j} }}^{2} + \sigma_{{e_{j} }}^{2} } \right)} }}$$

where $\sigma_{{f_{i} }}^{2}$ is the estimated family variance for trait $i$, and $\sigma_{{f_{j} }}^{2}$ is the estimated family variance for trait $j$. The difference between the mean breeding values of selected top ratio leaf traits and the total mean of the leaf trait was calculated as realized genetic gain ($\Delta G_{R}$).

## Data Availability

Not applicable.

## Abbreviations

PLSR: partial least squares regression
R^2^: coefficient of determination
${\text{R}}^{2}_{\text{CV}}$: coefficient of determination for cross-validation
${\text{R}}^{2}_{\text{V}}$: coefficient of determination for validation
RMSE: root mean square error
RMSE_CV_: cross-validation
RMSE_V_: root mean square error of validation
V_N_: selected variable numbers
${\text{R}}^{2}_{\text{M}}$: the mean value of coefficient of determination (R^2^) from 100 simulated models
RMSE_M_: root-mean-square error (RMSE) from 100 simulated models
sMC: significant Multivariate Correlation
*h*^2^: heritability
CC: chlorophyll content
